# RNA-seq analysis of synovial fibroblasts brings new insights into rheumatoid arthritis

**DOI:** 10.1186/2045-3701-2-43

**Published:** 2012-12-21

**Authors:** Daniel P Heruth, Margaret Gibson, Dmitry N Grigoryev, Li Qin Zhang, Shui Qing Ye

**Affiliations:** 1 Department of Pediatrics, Children's Mercy Hospitals and Clinics, University of Missouri School of Medicine, 2401 Gillham Road, Kansas City, MO, 64108, USA; 2 Department of Biomedical and Health Informatics, Children's Mercy Hospitals and Clinics, University of Missouri School of Medicine, 2401 Gillham Road, Kansas City, MO, 64108, USA

**Keywords:** RNA-seq, Next generation sequencing, Rheumatoid arthritis, Synovial fibroblasts, Transcriptional regulation

## Abstract

**Background:**

Rheumatoid arthritis (RA) is a chronic autoimmune-disease of unknown origin that primarily affects the joints and ultimately leads to their destruction. Growing evidence suggests that synvovial fibroblasts play important roles in the initiation and the perpetuation of RA but underlying molecular mechanisms are not understood fully. In the present study, Illumina RNA sequencing was used to profile two human normal control and two rheumatoid arthritis synvovial fibroblasts (RASFs) transcriptomes to gain insights into the roles of synvovial fibroblasts in RA.

**Results:**

We found that besides known inflammatory and immune responses, other novel dysregulated networks and pathways such as Cell Morphology, Cell-To-Cell Signaling and Interaction, Cellular Movement, Cellular Growth and Proliferation, and Cellular Development, may all contribute to the pathogenesis of RA. Our study identified several new genes and isoforms not previously associated with rheumatoid arthritis. 122 genes were up-regulated and 155 genes were down-regulated by at least two-fold in RASFs compared to controls. Of note, 343 known isoforms and 561 novel isoforms were up-regulated and 262 known isoforms and 520 novel isoforms were down-regulated by at least two-fold. The magnitude of difference and the number of differentially expressed known and novel gene isoforms were not detected previously by DNA microarray.

**Conclusions:**

Since the activation and proliferation of RASFs has been implicated in the pathogenesis of rheumatoid arthritis, further in-depth follow-up analysis of the transcriptional regulation reported in this study may shed light on molecular pathogenic mechanisms underlying synovial fibroblasts in arthritis and provide new leads of potential therapeutic targets.

## Background

Rheumatoid arthritis [RA] is a chronic, systemic autoimmune disorder associated with both genetic and environmental factors. RA affects 1% of the world’s population, develops most commonly in adults between 40 – 70 years old, and occurs more frequently in women than in men [1-4]. xAlthough the etiology of the disease has not been elucidated fully, the pathogenesis of RA is characterized by the influx of cells from both the innate and the adaptive immune systems [5]. These cells induce increased pro-inflammatory cytokine production, decreased synthesis of anti-inflammatory cytokines, and the subsequent activation and proliferation of synovial fibroblasts (SFs) [3,4]. Rheumatoid arthritis synovial fibroblasts (RASFs) produce additional cytokines, chemokines and matrix-degrading enzymes which ultimately leads to the thickening and progressive destruction of joint membrane, cartilage and bone [5-7]. Characterization of the cytokine signaling pathways involved in RA has provided a significant opportunity for identifying pro-inflammatory cytokines which can be targeted for novel therapeutic intervention. The development of biological response modifiers (BRMs), particularly the TNF, IL-1, and IL-6 antagonists, have led to major advances in RA therapy [3,7]. However, these agents are not effective in all patients, underscoring the genetic heterogeneity of the disease and the need for the development of additional BRMs [8]. RASFs are intricately involved in the pathogenesis of RA and provide a source for the identification of new genes and pathways that can be targeted for therapeutic intervention.

With the advent of next generation DNA sequencing technologies [9], such as RNA sequencing (RNA-seq), a more comprehensive and accurate transcriptome analysis has become feasible and affordable. In RNA-seq, short fragments of complementary DNA (cDNA) are sequenced (reads) and then mapped onto the reference genome. RNA-seq enables not only the identification of differentially expressed genes, but also the precise quantitative determination of exon and isoform (alternative splicing) expression, along with the characterization of transcription initiation sites (TSSs) and new splicing variants [10]. In the present study, we performed a comprehensive transcriptome analysis of RNA from RASFs from two adult female RA patients and the SF RNA from two healthy female donors, using the RNA-seq technique. We found significant differences in the expression levels of both genes and gene isoforms between normal SFs and RASFs RNA samples. These data provide broader and deeper insights, particularly with respect to isoform expression, into the effect of RA on the transcriptional regulation of synovial fibroblasts and a rich resource for further experimentation into the pathogenesis of the disease.

## Results

### RNA sequencing

Human SFs RNAs from two healthy control donors and two patients with RA were purchased from Cell Applications, Inc. (San Diego, CA). Diseased samples were age and sex-matched with normal controls (Additional file 1). Paired-end cDNA libraries for each RNA sample were prepared and sequenced using the Illumina TruSeq RNA Sample Preparation Kit, as outlined previously [11,12].

### Quality analysis of RNA-seq data

Real-time analysis of the sequencing run was performed by the Illumina HiSeq Control Software. Clusters of identical sequences were generated on the Illumina cBot and the number of those clusters was reported, along with the percentage of those clusters passing an internal quality filter. Across the 4 samples, between 433,000 and 482,000 raw clusters were detected, with a median of 446,000 clusters per lane. Between 90.9% and 95.0% of those clusters passed the filter, with a median of 93.2% of the clusters passing the filter. Each lane was aligned in real-time with the phiX genome and between 0.80% and 0.84% of the clusters aligned, with a median of 0.81% aligned. Our control lane of phiX produced 290,000 clusters with 97.9% passing the filter and 99.08% aligning to the phiX genome. All these values were within the recommended limits established by Illumina.

**Figure 1:**
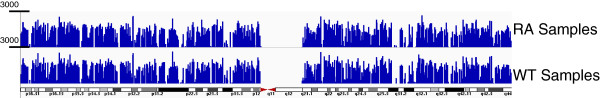
**A transcription profile of RNA from control synovial fibroblasts and rheumatoid arthritis fibroblasts for chromosome 1.** The RNA-seq read density plotted along chromosome 1 is shown. Average alignment was computed by igvtools. Each bar represents the log_2_ frequency of reads along the chromosome which range from 0 to 3000 for both control synovial fibroblasts and rheumatoid arthritis fibroblasts.

Post-run quality analysis of RNA-seq data was carried out as described by Twine et al. [13]. The total number of reads produced from each sample was between 80,782,262 and 89,757,726, with a mean across all samples of 84,177,268 (Table 1). The difference in the number of reads between the control samples and the RA samples was not statistically significant (Student’s t-test, p=0.27). To assess the quality of the reads, data was pulled from the TopHat log files as well as the output files. Between 0.10% and 0.15% of the reads were removed due to low quality before mapping to the reference genome began. Between 82.8% and 89.1% of the total reads mapped to the human genome. To ensure the uniform coverage across the genome, the data was visualized using a local copy of the Integrative Genomics Viewer. An example of the reads for both normal and RA patient samples mapped against chromosome 1 is shown in Figure 1. The average alignment was computed across the genome and those alignment scores were log-transformed (base 2) to better visualize the full range of the data. As expected, no reads mapped to the centromere or areas of the chromosome without genes.

**Table 1:** RNA-seq sequence reads mapping to UCSC Human genome build 19 by TopHat v1.3.0/Bowtie v0.12.7

	**WT**			**RA**		
	**1**	**2**	Average	**1**	**2**	Average
Total reads	80,782,262	82,738,536	81,760,399	89,757,726	83,430,548	86,594,137
Reads removed	0.10%	0.12%	0.11%	0.15%	0.12%	0.13%
Read aligned toreference genome	82.8%	84.6%	83.7%	89.1%	87.6%	88.4%

Total reads and the percentage of those reads removed due to low quality and aligned to hg19 by TopHat. TopHat allows two mismatches when aligning to a reference genome.

### Differentially expressed genes and isoforms

After mapping the sequencing reads to the reference genome with TopHat, transcripts were assembled and their relative expression levels were calculated with Cufflinks in *Fragments Per Kilobase of exon per Million fragments mapped* (FPKM). The sub-program, Cuffdiff was then used to calculate the differential expression on the gene and transcript level, as well as the calculation of alternative promoter usage and alternative splicing. Cufflinks calculates the differential gene expression with the ratio of the RA group to the control group for every gene and transcript along with the statistical significance of the values. Two categories of differential gene/isoform expression were identified. The first category consists of genes/isoforms expressed only in control SFs or only in RASFs. The second category consists of genes/isoforms in which expression of both samples in each group was up-regulated or down-regulated two-fold or greater between control SFs and RASFs.

Overall, there are 12,977 expressed genes in the control SFs and 13,445 expressed genes in the RASFs, which were aligned to the reference genome (Table 2). There are 214 genes, whose expressions were only detected in the normal SFs, while 682 genes whose expressions were only detected in RASFs. There are 122 up-regulated and 155 down-regulated genes in RASFs with at least two-fold change compared to the SFs (Table 2). As for known isoforms, there are 20,647 in the normal SFs and 21,102 in RASFs. Among them, there are 526 known isoforms, whose expressions were detected only in the normal SFs, while 981 known isoforms whose expressions were detected only in RASFs. There are 343 up-regulated and 262 down-regulated known isoforms in RASFs by at least two-fold change compared to the SFs (Table 2). For novel isoforms whose annotations are not known in the current reference gene or transcript database, there are 42,124 expressed in the normal SFs and 42,171 expressed in RASFs. Among them, there are 105 novel isoforms whose expressions were only detected in the normal SFs, while 152 novel isoforms were only detected in RASFs. There are 561 up-regulated and 520 down-regulated novel isoforms in RASFs by at least two-fold change compared to the SFs (Table 2).

**Table 2:** Gene/isoform expression summary

	**Genes**	
	Control	RA Patients
Total Genes Expressed	12,977	13,445
Control Only	214	
RA Patients Only		682
Up-regulated (2-fold or greater difference)		122
Down-regulated (2-fold or greater difference)		155
	**Known Isoforms**	
	Control	RA Patients
Total Known Isoforms Expressed	20,647	21,102
Control Only	526	
RA Patients Only		981
Up-regulated (2-fold or greater difference)		343
Down-regulated (2-fold or greater difference)		262
	**Novel Isoforms**	
	Control	RA Patients
Total Novel Isoforms Expressed	42,124	42,171
Control Only	105	
RA Patients Only		152
Up-regulated (2-fold or greater difference)		561
Down-regulated (2-fold or greater difference)		520

Genes, known isoforms and novel isoforms expressed in control synovial fibroblasts and synovial fibroblasts from patients with rheumatoid arthritis. Expression determined by Cufflinks, after normalization to a panel of housekeeping genes. The fold change is the ratio of RA FPKM to WT FPKM.

### Genes expressed only in control SFs or only in RASFs

The top 10 up- and down-regulated genes expressed only in control SFs or only in RASFs are presented in Table 3. An expanded list of the top 50 genes expressed only in either control SFs or in RASFs is presented in Additional file 2. Analysis of the genes expressed only in RASF reveals that nine of the top ten genes, including the major histocompatibility complex (MHC) genes HLA-A. –B, -C, and –E, are located on chromosome 6 (Table 3). Remarkably, 36 of the top 50 genes (Additional file 2) expressed only in RASFs are located on chromosome 6. The MHC, particularly the HLA-DRB1 alleles are strongly associated with RA [14-16]. A recent study by Plenge et al. has also identified associations of alleles lying outside the MHC on chromsome 6 with RA [17]. Our observation that the CLIC1 gene (chloride intracellular protein) is expressed in RASFs correlates with the finding that CLIC1(-/-) mice were protected from development of serum transfer induced K/BxN arthritis [18]. Two genes, the high mobility group box 1 (HMGA1) and the latent transforming growth factor beta binding protein 1 (LTBP1) have been reported to be elevated in RA [19,20] however they are not expressed in the RASFs examined in this study (Table 3). Interestingly, HMGA1 is the only gene on chromosome 6 in the list of top 50 genes expressed in normal SFs but not expressed in RASFs (Additional file 2). The CD59 complement regulatory protein (CD59) is not expressed in RASFs in this study. This observation supports the finding that CD59 is protective as CD59 (-/-) knockout mice present with more severe symptoms in the murine antigen-induced arthritis model [21]. An automated literature search using PubMatrix [22] reveals that eleven of the twenty genes listed in Table 3 have not yet been identified to be associated with RA (Additional file 3). These genes, which include chromosome 6 open reading frame 48 (C6orf48), the scavenger receptor class A, member 5 (SCARA5), CutA divalent cation tolerance homolog (CUTA), Leucine rich repeat containing 59 (LRRC59), and the protein phosphatase 1, regulatory (inhibitor) subunit 14A (PPP1R14A), may provide additional therapeutic targets. These potential targets include characterized genes, like the iron receptor SCARA5 [23] and genes, such as C6orf48, that have not yet been well-studied. CutA, which is up-regulated in RASFs, interacts with BACE1 to regulate B-cleavage of the B-amyloid protein (APP) [24]. CutA may play a role in the pathogenesis of Alzheimer’s, however, its role in rheumatoid arthritis remains to be elucidated. LRRC59 is required for the nuclear transport of the fibroblast growth factor 1 (FGF1) [25]. The affect on FGF1 function resulting from decreased LRRC59 expression in RASFs warrants further investigation. PPP1R14A, which inhibits protein phosphatase 1 activity, is not expressed in RASFs compared to normal SFs, suggesting that PP1 activity will increase dramatically in RASFs. PP1 controls the Akt signal transduction pathway to regulate cell growth, cell survival, and cell differentiation [26].

**Table 3:** Top ten up- and down- regulated genes expressed only in normal synovial RNA or only in rheumatoid arthritis synovial RNA

**Gene**	**Description**	**Chr**	**RA FPKM**	**RA2 FPKM**	**WT1 FPKM**	**WT2 FPKM**	**Avg.RA**	**Avg.WT**	**Ensembl gene ID**
HLA-B	Major histocompatibility complex, class 1, B	chr6	704.3	728.3	--	--	716.3	--	ENSG00000228964
HLA-A	Major histocompatibility complex, class 1, A	chr6	778.2	585.6	--	--	681.9	--	ENSG00000223980
HLA-C	Major histocompatibility complex, class 1, C	chr6	534.8	452.1	--	--	493.5	--	ENSG00000206435
TUBB	Tubulin, beta class I	chr6	405.3	416.7	--	--	411	--	ENSG00000232421
CLIC1	Chloride intracellular channel 1	chr6	350.5	369.6	--	--	360	--	ENSG00000223639
RPS18	Ribosomal Protein S18	chr6	260.4	269.1	--	--	264.8	--	ENSG00000227794
HLA-E	Major histocompatibility complex, class 1, E	chr6	243.5	260.2	--	--	251.9	--	ENSG00000230254
C6orf48	Chromosome 6 open reading frame 48	chr6	119.5	225.8	--	--	172.6	--	ENSG00000206380
SCARA5	Scavenger receptor class A, member 5	chr8	11.36	316	--	--	163.7	--	ENSG00000168079
CUTA	CutA divalent cation tolerance homolog	chr6	168.7	133.7	--	--	151.2	--	ENSG00000226492
ACTG2	Actin, gamma 2, smooth muscle, enteric	chr2	--	--	1087.76	2.58	--	545.17	ENSG00000163017
RPS24	Ribosomal Protein S24	chr10	--	--	407.72	429.08	--	418.40	ENSG00000138326
PSAP	Prosaposin	chr10	--	--	236.86	519.83	--	378.35	ENSG00000197746
HMGA1	High mobility group box 1	chr6:	--	--	139.35	265.81	--	202.58	ENSG00000189403
CD59	CD59 molecule, complement regulatory protein	chr11	--	--	111.47	149.28	--	130.38	ENSG00000085063
LRRC59	Leucine rich repeat containing 59	chr17	--	--	116.95	88.57	--	102.76	ENSG00000108829
PPP1R14A	Protein phosphatase 1, regulatory (inhibitor) subunit 14A	chr19	--	--	142.49	1.10	--	71.79	ENSG00000167641
LTBP1	Latent transforming growth factor beta binding protein 1	chr2	--	--	54.38	66.08	--	60.23	ENSG00000049323
SNHG6	Small nucleolar RNA host gene 6	chr8	--	--	46.68	65.62	--	56.15	ENSG00000245910
HNRNPC	Heterogeneous nuclear ribonucleoprotein C (C1/C2)	chr14	--	--	52.08	58.46	--	55.27	ENSG00000092199

Genes which were differentially expressed as determined by Cufflinks, after normalization to a panel of housekeeping genes.

The genes were ranked by FPKM and the 10 with the highest or lowest values are listed here.

### Genes differentially expressed two-fold or greater between control SFs and RASFs

The top 10 up- and down-regulated genes, along with the expanded top 50 list, in which expression of both samples in each group was up-regulated or down-regulated two-fold or greater between control SFs and RASFs are presented in Table 4 and in Additional file 4, respectively. Three genes in the top 10 up-regulated list have been associated with rheumatoid arthritis (Additional file 3). Interleukin 26 (IL26) is up-regulated (80.8-fold) in RASFs compared to SFs. Corvaisier et al. has demonstrated that IL26 is over-expressed in arthritis and induces inflammatory cytokine production [27]. The v-maf musculoaponeurotic fibrosarcoma oncogene homolog B (avian) (MAFB) gene is up-regulated (16.2-fold) in RASFs. Liu et al. identified polymorphisms in the MAFB gene associated with altered response to anti-TNF treatment in patients with RA [28]. Expression of the adrenergic, alpha-2A-, receptor (ADRA2A) increased 14.4-fold in RASFs. The adrenergic, alpha-2A-, receptor may play a critical role in the proliferation and differentiation of synoviocytes [29]. Although thrombospondin 4 (THSB4) has not yet been associated with arthritis (Additional file 3), thrombospondin 1 (THBS1) is over-expressed in RA tissue [30]. Thrombospondin 1 and 4 are extracellular matrix remodeling proteins that have been associated with increased inflammation in coronary artery disease (CAD) [30,31], and thus may provide a link between RA and CAD. Like THBS4, the remaining six genes up-regulated in RASFs (Table 4) have not yet been associated with RA but provide potential for further investigation. The solute carrier family members, SLC2A5, SLC14A1, and SLC12A8 are over-expressed in RASFs suggesting alterations in cellular metabolism. Complement Factor 1 (CF1) may represent a new target as the complement system plays a major role in the pathogenesis of rheumatoid disease [32]. Expression of the plasminogen activator inhibitor gene, serpin peptidase inhibitor, clade B (ovalbumin), member 2 (SERPINB2) is decreased (-79.1-fold) in RASFs compared to control SFs (Table 4). The plasminogen activation pathway is dysregulated in arthritis [33]. Aquoporin 1 (AQP1) expression has been shown to be up-regulated in the synovium from RA patients [34], but is down-regulated (-44.3-fold) in our samples. The coagulation factor X (F10), which may contribute to tissue injury and remodeling [35], is down-regulated (-27.5 fold). The hedgehog interacting protein (HHIP) inhibits the sonic hedgehog (SSH) signaling pathway. Inactivation of SSH inhibitor smoothened (Smo) blocks sonic hedgehog signaling and prevents osteophyte formation in the murine serum transfer arthritis model [36]. Thus, the decrease (-26.6-fold) in HHIP expression observed in RASFs in this study may result in increased SSH activity resulting in advanced osteophyte formation.

**Table 4:** Top ten up- and down- regulated genes expressed in rheumatoid arthritis synovial RNA

**Gene**	**Description**	**Chr**	**RAFPKM**	**RA2FPKM**	**WT2FPKM**	**WT2FPKM**	**Avg. RA**	**Avg. WT**	**Fold change**	**Ensembl gene ID**
IL26	interleukin 26 solute carrier family 2 (facilitated	chr12	17.913	1.927	0.101	0.144	9.920	0.123	80.83	ENSG00000111536
SLC2A5	glucose/fructose transporter), member 5	chr1	65.268	21.844	0.340	3.851	43.556	2.096	20.79	ENSG00000142583
PLXDC2	plexin domain containing 2 v-maf musculoaponeurotic	chr10	9.222	2.316	0.098	0.573	5.769	0.335	17.21	ENSG00000120594
MAFB	fibrosarcoma oncogene homolog B (avian) solute carrier family 14 (urea	chr20	37.177	6.679	0.605	2.096	21.928	1.351	16.24	ENSG00000204103
SLC14A1	transporter), member 1 (Kidd blood group	chr18	6.188	2.096	0.360	0.177	4.142	0.269	15.41	ENSG00000141469
ADRA2A	adrenergic, alpha-2A-, receptor	chr10	4.373	10.666	0.899	0.145	7.519	0.522	14.42	ENSG00000150594
MAN1C1	mannosidase, alpha, class 1C, member 1	chr1	16.774	24.3346	0.65654	2.7991	20.554	1.728	11.90	ENSG00000117643
CFI	complement factor I solute carrier family 12	chr4	21.803	28.7589	0.10437	4.3263	25.281	2.215	11.41	ENSG00000205403
SLC12A8	(potassium/chloride transporters), member 8	chr3	7.442	15.2539	0.62851	1.4395	11.348	1.034	10.97	ENSG00000221955
THBS4	thrombospondin 4	chr5	5.8622	7.09226	0.05843	1.2202	6.477	0.639	10.13	ENSG00000113296
SERPINB2	serpin peptidase inhibitor, clade B (ovalbumin), member 2	chr18	0.236	0.095	16.207	10.030	0.166	13.118	−79.11	ENSG00000197632
AQP1	aquaporin 1 (Colton blood group)	chr7	5.675	3.860	396.183	25.802	4.768	210.992	−44.26	ENSG00000240583
APOBEC3B	apolipoprotein B mRNA editing enzyme, catalytic polypeptide-like 3B	chr22	0.0775	0.23398	2.59902	7.4102	0.156	5.005	−32.13	ENSG00000179750
NEFM	neurofilament, medium polypeptide	chr8	0.0592	0.07122	3.69528	0.2014	0.065	1.948	−29.87	ENSG00000104722
CCDC3	coiled-coil domain containing 3	chr10	0.1241	0.09255	4.48673	1.7074	0.108	3.097	−28.59	ENSG00000151468
F10	coagulation factor X	chr13	0.197	0.21727	8.44739	2.9581	0.207	5.703	−27.53	ENSG00000126218
HHIP	hedgehog interacting protein	chr4	0.073	0.26532	7.88577	1.1075	0.169	4.497	−26.58	ENSG00000164161
ARL2-SNX15	-	chr11	0.361	0.39062	8.29552	11.168	0.376	9.732	−25.90	-
HES4	hairy and enhancer of split 4	chr1	0.3015	0.46829	16.2396	1.0894	0.385	8.664	−22.51	ENSG00000188290
GPAT2	glycerol-3-phosphate acyltransferase 2, mitochondrial	chr2	0.5547	0.36585	17.6005	3.0486	0.460	10.325	−22.43	ENSG00000186281

Genes which were differentially expressed as determined by Cufflinks, after normalization to a panel of housekeeping genes. The fold change is the ratio of RASF FPKM to control FPKM. Genes with a fold change of 1.2-fold or greater were defined as significant. The genes were ranked on their fold change and the 10 with the highest or lowest fold changes are listed here.

### Known isoforms expressed only in control SFs or only in RASFs

The top 10 isoforms expressed only in control SFs or only in RASFs are presented in Table 5. An expanded list of the top 50 up- and down-regulated known isoforms expressed only in either control SFs or in RASFs is presented in Additional file 5. The known isoforms identified in Table 5 correlate with the genes expressed only in control SFs or only in RASFs (Table 3 and Additional file 2). Single isoforms were detected for SCARA5, PLA2G2A, SPCS1, CITED2, IL13RA2, SLP1, FAM20A, NUMA1, PSAP, LRRC59, PPP1R14A, and SNHG6. Two isoforms were identified for PRG4, ACTG2, and CD59, while five and six isoforms exist for RPS24 and HMG1A, respectively (Table 5 and Additional file 5).

**Table 5:** Top ten up- and down- regulated isoforms expressed only in normal synovial RNA or only in rheumatoid arthritis synovial RNA

**Gene**	**Description**	**Locus**	**Length**	**RA1FPKM**	**RA2FPKM**	**WT1FPKM**	**WT2FPKM**	**Avg. RA**	**Avg. WT**	**Ensembl gene ID**
SCARA5	Scavenger receptor class A, member 5	chr8	4151	11.36	353.51	--	--	182.43	--	ENSG00000168079
PLA2G2A	Phopholipase A2, group IIA	chr1	969	3.81	264.84	--	--	134.33	--	ENSG00000188257
SPCS1	Signal peptidase complex subunit 1 homolog	chr3	1084	81.32	112.54	--	--	96.93	--	ENSG00000114902
CITED2	Cbp/p300-interacting transactivator, 2	chr6	1929	69.03	119.73	--	--	94.38	--	ENSG00000164442
IL13RA2	Interleukin 13 receptor, alpha 2	chrX	1373	9.94	90.27	--	--	50.10	--	ENSG00000123496
SLPI	Secretory leukocyte peptidase inhibitor	chr20	598	1.36	98.75	--	--	50.06	--	ENSG00000124107
KYNU	Kynureninase	chr2	1672	16.46	79.31	--	--	47.88	--	ENSG00000115919
FAM20A	Family with sequence similarity 20, member A	chr17	4275	28.98	60.54	--	--	44.76	--	ENSG00000108950
NUMA1	Nuclear mitotic apparatus protein 1	chr11	7182	40.51	45.42	--	--	42.96	--	ENSG00000137497
PRG4	Proteoglycan 4	chr1	4765	1.27	81.59	--	--	41.43	--	ENSG00000116690
ACTG2	Actin, gamma 2, smooth muscle, enteric	chr2	1331	--	--	1046.45	2.16	--	524.30	ENSG00000163017
PSAP	Prosaposin	chr10	2822	--	--	234.56	510.86	--	372.71	ENSG00000197746
RPS24	Ribosomal Protein S24	chr10	655	--	--	149.70	298.29	--	224.00	ENSG00000138326
LRRC59	Leucine rich repeat containing 59	chr17	2915	--	--	116.95	88.57	--	102.76	ENSG00000108829
HMGA1	High mobility group box 1	chr6	1846	--	--	44.78	99.59	--	72.19	ENSG00000189403
CD59	CD59 molecule, complement regulatory protein	chr11	7619	--	--	68.61	75.31	--	71.96	ENSG00000085063
PPP1R14A	Protein phosphatase 1, regulatory (inhibitor) subunit 14A	chr19	718	--	--	142.49	1.10	--	71.79	ENSG00000167641
HMGA1	High mobility group box 1	chr6	1993	--	--	43.81	93.26	--	68.54	ENSG00000189403
RPS24	Ribosomal Protein S24	chr10	633	--	--	80.53	47.31	--	63.92	ENSG00000138326
SNHG6	Small nucleolar RNA host gene 6	chr8	472	--	--	46.68	65.62	--	56.15	ENSG00000245910

Isoforms which were differentially expressed as determined by Cufflinks, after normalization to a panel of housekeeping genes.

The isoforms were ranked by FPKM and the 10 with the highest or lowest values are listed here.

### Known isoforms differentially expressed two-fold or greater between control SFs and RASFs

The top 10 up- and down-regulated known isoforms, along with the expanded top 50 list, in which expression of both samples in each group was up-regulated or down-regulated two-fold or greater between control SFs and RASFs are presented in Table 6 and in Additional file 6, respectively. Thirteen of the known isoforms identified in Table 6 can be found in the top 50 up-regulated and down-regulated genes presented in Table 4 and Additional file 4. A single isoform of IL26 is expressed 80.8-fold and correlates with the expression (80.8-fold) of the IL26 gene in RASFs. Seven known isoforms (ILI27, DHPS, BLCAP, LYNX1, C5orf13, APLP2, and CSRP1) are not represented in the top 50 regulated genes. One reason for this observation is differential isoform expression, as demonstrated by the two isoforms of Interferon, alpha-inducible protein 27 (ILI27). One ILI27 isoform is up-regulated 35.8-fold and one is down-regulated 216.8-fold. Two known isoforms were also identified for GCNT1, SLC2A5 and C5orf13 in the top 50 list.

**Table 6:** Top ten up- and down- regulated known isoforms expressed in rheumatoid arthritis synovial RNA

**Gene**	**Description**	**Locus**	**Length**	**RA1FPKM**	**RA2FPKM**	**WT1FPKM**	**WT2FPKM**	**Avg. RA**	**Avg. WT**	**Fold change**	**Ensembl gene ID**
IL26	interleukin 26	chr12	1047.00	17.91	1.93	0.10	0.14	9.92	0.12	80.83	ENSG00000111536
GCNT1	glucosaminyl (N-acetyl) transferase 1, core 2	chr9	5478.00	3.99	3.35	0.08	0.05	3.67	0.07	55.82	ENSG00000187210
IFI27	interferon, alpha-inducible protein 27	chr14	652.00	272.04	223.93	5.09	8.77	247.99	6.93	35.79	ENSG00000165949
GCNT1	glucosaminyl (N-acetyl) transferase 1, core 2	chr9	5596.00	3.81	2.20	0.06	0.13	3.01	0.09	32.54	ENSG00000187210
IGFBP3	insulin-like growth factor binding protein 3	chr7	2631.00	123.09	213.34	2.30	8.88	168.22	5.59	30.09	ENSG00000146674
DHPS	deoxyhypusine synthase	chr19	1184.00	12.04	2.92	0.40	0.14	7.48	0.27	27.42	ENSG00000095059
BLCAP	bladder cancer associated protein	chr20	2073.00	9.25	2.16	0.20	0.32	5.70	0.26	22.14	ENSG00000166619
SLC2A5	solute carrier family 2 (facilitated glucose/fructose transporter), member 5	chr1	2438.00	62.93	20.78	0.21	3.63	41.85	1.92	21.79	ENSG00000142583
SLC12A8	solute carrier family 12 (potassium/chloride transporters), member 8	chr3	3447.00	6.34	16.64	0.32	0.73	11.49	0.52	22.01	ENSG00000221955
LYNX1	Ly6/neurotoxin 1	chr8	1290.00	6.07	3.23	0.39	0.06	4.65	0.23	20.48	ENSG00000180155
C5orf13	chromosome 5 open reading frame 13	chr5	1996.00	0.32	0.71	303.46	6.35	0.52	154.91	−300.00	ENSG00000134986
IFI27	interferon, alpha-inducible protein 27	chr14	648.00	0.40	1.10	5.62	318.85	0.75	162.23	−216.80	ENSG00000165949
C5orf13	chromosome 5 open reading frame 13	chr5	2068.00	0.14	0.19	40.42	1.50	0.16	20.96	−127.90	ENSG00000134986
APLP2	amyloid beta (A4) precursor-like protein 2	chr11	3274.00	0.06	0.23	5.27	15.27	0.14	10.27	−71.07	ENSG00000084234
CSRP1	cysteine and glycine-rich protein 1	chr1	1938.00	0.20	0.41	34.33	3.74	0.31	19.03	−61.54	ENSG00000159176
AQP1	aquaporin 1	chr7	2807.00	4.71	2.55	390.29	23.23	3.63	206.76	−56.95	ENSG00000240583
PARP2	poly (ADP-ribose) polymerase 2	chr14	1887.00	0.10	0.19	2.43	6.85	0.14	4.64	−32.85	ENSG00000129484
APOBEC3B	apolipoprotein B mRNA editing enzyme, catalytic polypeptide-like 3B	chr22	1536.00	0.08	0.26	2.60	7.41	0.17	5.00	−29.50	ENSG00000179750
CCDC3	coiled-coil domain containing 3	chr10	2738.00	0.12	0.10	4.49	1.71	0.11	3.10	−27.21	ENSG00000151468
MTIF3	mitochondrial translational initiation factor 3	chr13	1098.00	0.14	0.22	3.98	5.73	0.18	4.85	−26.92	ENSG00000122033

Isoforms which were differentially expressed as determined by Cufflinks, after normalization to a panel of housekeeping genes. The fold change is the ratio of RASF FPKM to control FPKM. Isoforms with a fold change of 1.2-fold or greater were defined as significant. The isoforms were ranked on their fold change and the 10 with the highest or lowest fold changes are listed here.

### Novel isoforms expressed only in control SFs or only in RASFs

The top 10 up- and down-regulated novel isoforms expressed only in control SFs or only in RASFs are presented in Table 7. An expanded list of the top 50 up- and down-regulated known isoforms expressed only in either control SFs or in RASFs is presented in Additional file 7. The list of the top 10 up-regulated novel isoforms includes transcripts for four unannotated genomic regions. The top 50 novel isoforms contains 21 transcripts from unannotated genomic regions. The list of top 10 down-regulated novel isoforms is divided into nine isoforms from annotated genes, including a novel transcript for HHIP, and one down-regulated novel isoform. There are transcripts for fourteen unannotated genomic regions in the top 50 down-regulated novel isoforms.

**Table 7:** Top ten up- and down- regulated novel isoforms expressed only in normal synovial RNA or rheumatoid arthritis synovial RNA

**Gene**	**Description**	**Coordinates**	**Length**	**FPKM Wildtype**	**FPKM RA**	**Ensembl gene ID**
GIPC1	GIPC PDZ domain containing family. Member 1	chr19:14588570-14606944	1650	--	8.09135	ENSG00000123159
MPPE1	Metallophosphoesterase 1	chr18:11883385-11908455	1973	--	5.66007	ENSG00000154889
-	NA	chr11:69066649-69184402	410	--	5.19725	NA
EPB41L2	Erythrocyte membrane protein band 4.1-like 2	chr6:131160487-131384462	3393	--	4.45046	ENSG00000079819
MRPL14	Mitochondrial ribosomal protein L14	chr6:44072507-44123256	658	--	4.29538	ENSG00000180992
PPIEL	Peptidylprolyl isomerase E-like pseudogene	chr1:39987953-40025316	509	--	4.21536	ENSG00000243970
-	NA	chr6:166822859-167041186	3525	--	3.83632	NA
-	NA	chr21:39607975-39679370	1369	--	3.3631	NA
FAM101A	Family with sequence similarity 101. member A	chr12:124774147-124800566	2242	--	3.16322	ENSG00000178882
-	NA	chr4:39454172-39460535	666	--	3.12642	NA
-	NA	chr20:30432079-30433458	1379	18.1953	--	NA
GPAT2	Glycerol-3-phosphate acyltransferase 2, mitochondrial	chr2:96687342-96700658	2732	6.12103	--	ENSG00000186281
PCDHGC5	Protocadherin gamma subfamily C, 5	chr5:140746308-140914003	4930	6.08509	--	ENSG00000240764
RSAD2	Radical S-adenosyl methionine domain containing 2	chr2:6988770-7038095	5210	4.87066	--	ENSG00000134321
HEYL	Hairy/enhancer of split related with YRPW motif-like	chr1:40089102-40105348	3872	4.34476	--	ENSG00000163909
GPR107	G protein-coupled receptor 107	chr9:132815745-132902440	3463	4.15184	--	ENSG00000148358
GOLGA2	Golgin A2	chr9:131018105-131038268	3014	2.8846	--	ENSG00000167110
HHIP	Hedgehog interacting protein	chr4:145567142-145660251	2628	2.57248	--	ENSG00000164161
ITIH3	Inter-alpha-trypsin inhibitor heavy chain 3	chr3:52828743-52838029	1944	2.20862	--	ENSG00000162267
HEATR5A	HEAT repeat containing 5A	chr14:31757730-31889797	6427	2.17675	--	ENSG00000129493

Novel isoforms which were differentially expressed as determined by CuffDiff after Benjamini-Hochberg correction. The isoforms were ranked by FPKM and the 10 with the highest or lowest fold changes are listed here.

### Novel isoforms differentially expressed two-fold or greater between control SFs and RASFs

The top 10 up- and down-regulated novel isoforms, along with the expanded top 50 list, in which expression of both samples in each group was up-regulated or down-regulated two-fold or greater between control SFs and RASFs are presented in Table 8 and in Additional file 8, respectively. A transcript of Fibrillin 1 (FBN1) is the top up-regulated novel isoform. Of note, a mutation in FBN1, which encodes an extracellular matrix glycoprotein, has been associated with the coexistence of Marfan’s Syndrome and ankylosing spondylitis [37]. Novel isoforms from three unannotated regions of the genome were identified in the top 10 up-regulated novel isoforms. A total of 13 novel isoforms identified within unannotated regions of the genome were up-regulated in RASFs compared to SFs (Additional file 8). The list of top 10 down-regulated novel isoforms is divided into nine isoforms from annotated genes and one down-regulated novel isoform. A total of 10 novel isoforms within unannotated regions of the genome were down-regulated in RASFs compared to SFs. Interestingly, there are two novel transcripts for both HLA-DRB1 and SLC2A5 identified in this study (Additional file 8).

**Table 8:** Top ten up- and down- regulated novel isoforms expressed in rheumatoid arthritis synovial RNA

**Gene**	**Description**	**Coordinates**	**Length**	**FPKM Wildtype**	**FPKM RA**	**Fold change**	**Ensembl gene ID**
FBN1	fibrillin 1	chr15:48700502-48944261	3642	0.35665	122.625	343.82	ENSG00000166147
TNXB	tenascin XB	chr6:31913771-32077409	10005	0.0711364	9.52612	133.91	ENSG00000168477
VCAN	versican	chr5:82767225-82878111	7388	0.145287	17.706	121.87	ENSG00000038427
LRP1	low density lipoprotein receptor-related protein 1	chr12:57522228-57607140	6609	0.223758	19.9154	89.00	ENSG00000123384
DPYSL2	dihydropyrimidinase-like 2	chr8:26435420-26515693	3416	0.287829	23.9348	83.16	ENSG00000092964
-	Genes nearby:FAM198B: family with sequence similarity 198, member B	chr4:159045731-159093718	1964	0.064901	5.20752	80.24	ENSG00000164125
-	Genes nearby:TGFBR3: transforming growth factor, beta receptor III	chr1:92145899-92351836	1323	0.137404	11.0015	80.07	ENSG00000069702
ALDH1L2	aldehyde dehydrogenase 1 family, member L2	chr12:105413561-105478341	4568	0.0522639	3.45172	66.04	ENSG00000136010
-	NA	chr14:74964883-75079368	2880	0.114262	6.82866	59.76	NA
	Genes nearby: ISCA2: iron-sulfur cluster assembly 2 homolog						ENSG00000165898
SNED1	LTBP2: latent transforming growth factor beta binding protein 2	chr2:241936998-242041710	8107	0.15755	9.34599	59.32	ENSG00000119681
TINAGL1	tubulointerstitial nephritis antigen-like 1	chr1:32041807-32053290	995	129.883	0.462813	−280.64	ENSG00000142910
TPM2	tropomyosin 2 (beta)	chr9:35681989-35690053	1083	78.6638	0.329924	−238.43	ENSG00000198467
MT2A	metallothionein 2A	chr16:56642376-56692994	248	701.232	5.35657	−130.91	ENSG00000125148
FSTL1	follistatin-like 1	chr3:120113060-120169918	1640	10.7318	0.0822263	−130.52	ENSG00000163430
ITPRIP	inositol 1,4,5-trisphosphate receptor interacting protein	chr10:106069730-106098576	6523	18.5495	0.146115	−126.95	ENSG00000148841
-	NA	chr13:41958154-41958844	690	51.6499	0.522452	−98.86	NA
SPTBN1	spectrin, beta, non-erythrocytic 1	chr2:54683453-54898583	7086	12.396	0.126624	−97.90	ENSG00000115306
HLA-DRB1	major histocompatibility complex, class II, DR beta 5	chr6:32441211-32557589	513	12.6351	0.129095	−97.87	ENSG00000198502
SEMA3F	sema domain, immunoglobulin domain (Ig), short basic domain, secreted, (semaphorin) 3F	chr3:50192454-50226507	3394	6.92733	0.0764133	−90.66	ENSG00000001617
CNN1	calponin 1, basic, smooth muscle	chr19:11649578-11661139	659	67.4653	0.799449	−84.39	ENSG00000130176

Novel isoforms which were differentially expressed as determined by CuffDiff after Benjamini-Hochberg correction. The fold change is the ratio of RASF FPKM to control FPKM. Novel isoforms with a fold change of 1.2-fold or greater were defined as significant. The isoforms were ranked on their fold change and the 10 with the highest or lowest fold changes are listed here.

### Network and pathway analyses of differentially expressed genes

To identify network and pathway connectivity, the differentially expressed gene lists of a two-fold or greater change in RASFs compared to SFs were submitted to Ingenuity Pathway Analysis (IPA) v9.0-3211 (Ingenuity Systems, Inc., Redwood City, CA), as described in the Material and Methods section. The networks affected by up-regulated genes and isoforms in RASFs compared to normal SFs are listed in Table 9. Consistent with the knowledge that RA is an immune disorder, the top network predicted to be affected by the up-regulated genes was *Inflammatory Response, Immunological Disease, Cell Death*, while the top network predicted to be affected by the up-regulated isoforms was *Inflammatory Response, Cellular Movement, Cell-To-Cell Signaling and Interaction*. The pathways affected by up-regulated genes and/or isoforms correlated with the pathways predicted to be affected by down-regulated gene expression and changes in isoform expression (Table 10). The top networks affected by down-regulated genes and isoforms in RASFs compared to normal SFs are *Cellular Movement, Cell Death, and Tissue Development* and *Cellular Growth and Proliferation, Cell Death, Cellular Movement*, respectively.

**Table 9:** Top networks affected by up-regulated genes/isoforms in rheumatoid arthritis synovial RNA

	**Up-regulated genes**	
Top Functions	Score	Genes
Inflammatory Response, Immunological Disease, Cell Death	68	58
Cell Morphology, Tissue Development, Cell Death	30	36
Cell-To-Cell Signaling and Interaction, Hematological System		
Development and Function, Immune Cell Trafficking	25	32
Inflammatory Response, Infectious Disease, Immunological Disease	23	31
Cellular Development, Cancer, Developmental Disorder	22	30
Inflammatory Response, Cellular Development, Cell Death	22	30
Cell Death, Hematological System Development and Function, Tissue Morphology	22	30
	Up-Regulated Isoforms	
Top Functions	Score	Genes
Inflammatory Response, Cellular Movement, Cell-To-Cell Signaling and Interaction	88	70
Cellular Development, Cell Death, Cellular Growth and Proliferation	26	36
Inflammatory Response, Organismal Injury and Abnormalities, Cellular Movement	24	35
Cellular Growth and Proliferation, Cellular Development, Cancer	24	35
Cell-To-Cell Signaling and Interaction, Inflammatory Response, Hematological System Development and Function	23	34
Cellular Movement, Hematological System Development and Function, Immune Cell Trafficking	23	34

Networks significantly affected in RASFs compared to control SFs as determined by Ingenuity Pathway Analysis. The score is based on the p-value of the affected network. Networks with a score of 15 or greater were defined as significant.

**Table 10:** Top networks affected by down-regulated genes/isoforms in rheumatoid arthritis synovial RNA

	**Down-regulated genes**	
Top Functions	Score	Genes
Cellular Movement, Cell Death, Tissue Development	35	32
Cellular Growth and Proliferation, Cellular Development, Hematological System Development and Function	29	28
Cell Cycle, Cellular Growth and Proliferation, Cell Death	27	27
Cellular Growth and Proliferation, Cell Cycle, Tissue Development	23	24
Hematological System Development and Function, Tissue Morphology, Tissue Development	21	23
	Down-Regulated Isoforms	
Top Functions	Score	Genes
Cellular Growth and Proliferation, Cell Death, Cellular Movement	45	43
DNA Replication, Recombination, and Repair, Cell Cycle, Hematological System Development and Function	28	32
Cellular Development, Cell Morphology, Cellular Assembly and Organization	25	30
Cellular Growth and Proliferation, Tissue Morphology, Hematological System Development and Function	25	30
Cellular Growth and Proliferation, Cellular Movement, Embryonic Development	24	29
Cell Death, Cellular Development, Hematological System Development and Function	24	29

Networks significantly affected in RASFs compared to control SFs as determined by Ingenuity Pathway Analysis. The score is based on the p-value of the affected network. Networks with a score of 15 or greater were defined as significant.

Canonical pathways analyses identified the pathways from the Ingenuity Pathways Analysis library of canonical pathways that were most significant to the data set. Genes with a two-fold or greater change in expression between SFs and RASFs and that were associated with a canonical pathway in Ingenuity’s Knowledge Base were considered for the analyses. The top canonical pathways affected by up-regulated genes and isoforms (Table 11) and the top canonical pathways affected by down-regulated genes and isoforms (Table 12) are in agreement with the networks (Tables 9 and 10) affected in RASFs. The top canonical pathways affected by up-regulated genes and isoforms (Table 11) are consistent with the knowledge that B cells, T cells, and macrophage cells play key roles in the inflammatory response and are involved in the activation and proliferation of RASFs [3,4,7]. These findings are further supported by the analysis of the pathways affected by the down-regulated genes and isoforms (Table 12). Dysregulation of the innate immune response and alterations in the number and types of cytokines and chemokines are well known features of RA [4,7]. Altered cell cycle control of chromosomal replication and BRCA1 in DNA damage response, are in concordance with the hyperproliferation of synovial tissue and the corresponding decrease in apoptosis in RA [3,38]. The identification of potential networks and pathways involved in arthritis may provide additional insights into the molecular and cellular mechanisms by which RASFs are involved in the pathogenesis of RA.

**Table 11:** Top canonical pathways affected by up-regulated genes/isoforms in rheumatoid arthritis synovial RNA

	**Up-regulated genes**	
Canonical Pathway	p-value	Ratio
Antigen Presentation Pathway	0.000	0.455
Graft-versus-Host Disease Signaling	0.000	0.275
Communication between Innate and Adaptive Immune Cells	0.000	0.188
Crosstalk between Dendritic Cells and Natural Killer Cells	0.000	0.159
Autoimmune Thyroid Disease Signaling	0.000	0.214
	Up-regulated Isoforms	
Canonical Pathway	p-value	Ratio
Atherosclerosis Signaling	0.001	0.133
Hepatic Fibrosis / Hepatic Stellate Cell Activation	0.001	0.119
Colorectal Cancer Metastasis Signaling	0.010	0.088
Toll-like Receptor Signaling	0.011	0.143
FXR/RXR Activation	0.012	0.114

Top canonical pathways significantly affected in RASFs compared to SFs as determined by Ingenuity Pathway Analysis. Pathways with a p-value less than 0.05 defined as significant.

**Table 12:** Top canonical pathways affected by down-regulated genes/isoforms in rheumatoid arthritis synovial RNA

	**Down-regulated genes**	
Canonical Pathway	p-value	Ratio
LXR/RXR Activation	0.011	0.057
Atherosclerosis Signaling	0.011	0.057
LPS/IL-1 Mediated Inhibition of RXR Function	0.017	0.044
Inhibition of Angiogenesis by TSP1	0.018	0.083
Phenylalanine Metabolism	0.019	0.086
	Down-regulated Isoforms	
Canonical Pathway	p-value	Ratio
Role of BRCA1 in DNA Damage Response	0.002	0.125
Mitotic Roles of Polo-Like Kinase	0.002	0.123
Cardiac β-adrenergic Signaling	0.004	0.079
Type I Diabetes Mellitus Signaling	0.005	0.086
Graft-versus-Host Disease Signaling	0.007	0.125

Top canonical pathways significantly affected in RASFs compared to SFs as determined by Ingenuity Pathway Analysis. Pathways with a p-value less than 0.05 defined as significant.

## Discussion

In the present study, we performed a comprehensive transcriptome analysis of human SF RNA isolated from healthy controls and patients with RA using the Illumina RNA-seq technique. It has revealed a complete picture of differentially expressed genes and their isoforms in RASFs and provided a global transcriptional insight into the novel roles of synovial fibroblasts in the pathogenesis of rheumatoid arthritis.

For RNA-seq, we used the Illumina HiScanSQ instrument to perform a 2 × 101 paired end run for all of our samples. The advantage of a paired end run is that both reads contain long range positional information, allowing for highly precise alignment of reads. We calculated the number of differentially expressed genes between RNA from two control SF and two RASF samples. We obtained a mean value of 84,177,268 reads per sample, which meets the criteria for sufficient sequence coverage for transcriptome profiling [39]. Our mean rate of 86% total reads that map to the reference genome met quality standards of the RNA-seq technique [40]. The breadth of the RNA sequencing reads covering chromosome 1 for both the RASFs and normal SFs indicates quality RNA-seq runs (Figure 1). Therefore, we are confident that our RNA-seq data provides an objective, high quality profile of the transcriptome in human RASFs and normal SFs.

The aim of this study was to provide a global glean into the transcriptional regulation in RASFs, which may provide mechanistic insights into the pathogenesis of rheumatoid arthritis. The activation and subsequent proliferation of SFs by proinflammatory cytokines produced by cells from both the innate and the adaptive immune systems plays a critical role in the pathogenesis of RA [3-5]. The production of additional cytokines, chemokines and matrix-degrading enzymes by RASFs leads ultimately to the progressive destruction of the joint that is a hallmark feature of RA [5-7]. However, the complete repertoire of active molecules, networks and pathways of differentially expressed genes and their isoforms of RASFs in this process are not characterized fully. Our study is filling this gap of knowledge. With RNA-seq, we found that 214 genes were not expressed in RASFs while 682 genes were only expressed in RASFs (Table 2). There are 122 up-regulated genes and 155 down-regulated genes by at least two-fold in RASFs compared to those in normal SFs. The majority of differentially expressed genes identified in this study (Tables 3 and 4 and Additional files 2 and 4) have not been previously reported to be altered in RASFs compared to normal SFs. One notable prowess of RNA-seq is to identify and quantify the expression of different isoforms of a gene. Gene isoforms are generated by alternative splicing or alternative promoter usage. Regulation of different gene isoform expression is a central aspect of most normal and disease processes. In this study, we detected more than 20,000 expressed known isoforms and more than 40,000 expressed novel isoforms (Table 2). Among them, there are 526 known isoforms which were not expressed in RASFs while 981 known isoforms were only expressed in RASFs. There are 343 up-regulated known isoforms and 262 down-regulated known isoforms by at least two-fold in RASFs compared to those in normal SFs. There are 105 novel isoforms which were not expressed in RASFs, while 152 novel isoforms were expressed only in RASFs. There are 561 up-regulated novel isoforms and 520 down-regulated novel isoforms by at least two-fold in RASFs compared to those in normal SFs. Network and canonical pathway analyses of differentially expressed genes and their known isoforms revealed that inflammatory response and cell death are represented strongly. Although these pathways have been predicted previously to correlate with RA, our study provided a more complete list of genes and isoforms involved in the inflammatory response and cell death pathways. We also identified other relevant novel networks and pathways, such as *Antigen Presentation Pathway*, *Atherosclerosis Signalling*, *LXR/RXR Activation*, and *Role of BRCA1 in DNA Damage Response*, whose dysregulation may each in part underlie their implication in the pathogenesis of RA.

Several microarray transcriptome analyses have been performed on RASFs [41-53]. The heterogeneous nature of RA and the different types of tissues used in these microarray studies leads to variations between the studies. The results from the present RNA-seq study both correlated and differed from previous microarray studies. The SFs used in our study were first isolated from synovial tissue either from healthy control donors or from patients with RA and cultured for two passages prior to RNA isolation. It should be noted, that this passage number is lower than what has been reported previously for gene profiling in SFs that have been cultured prior to RNA isolation. Del Rey et al. [43] and Masuda et al. [47] cultured SFs for 4 and 6 passages, respectively, before isolating RNA, while Haupl et al. [48] used immortalized SFs. The matrix metalloproteinases 1 (MMP1) and 3 (MMP3) are key players in the pathogenesis of RA [50]. MMP1 and MMP3 were up-regulated 816.2- fold and 215.6-fold, respectively, in our study. Microarray analyses of RA synovial tissue in three separate studies detected increased MMP1 expression of 63.1-fold [51], 31.0-fold [52], and 36.6-fold [53]. MMP3 expression was also increased 23.2-fold [52] and 18.7-fold [53] in these studies. Interleukin 1 beta (IL1B) and Interleukin 8 were up-regulated 3.2 and 9.3 fold, respectively, in RASFs from patients treated with prednisolone [48]. In the present study, IL1B was decreased by 25.3-fold and IL8 was down-regulated 9.5-fold. Collagen, Type III, alpha 1 (COL3A1) was increased 1.76 fold in a microarray study [44] compared to a 1.3-fold decrease in the present study. Keratin 7 (KRT7) was down-regulated 0.49 by microarray analysis [44] and 14.6-fold by RNA-seq. The results presented in our study correlate well with what has been previously reported in the literature. Of the top 40 differentially expressed genes (Tables 3 and 4), 16 have been reported previously to be associated with RA (Additional file 3). Thus, we have identified 24 new potential gene targets among the genes listed in Tables 3 and 4 for further exploration. These findings are strengthened further by the ability of RNA-seq, as described above, to identify isoforms, both known and novel, that are expressed differentially in RA. With further improvements of next generation DNA sequencing techniques and further reductions of sequencing costs, it may be feasible to extend this study to analyze the transcriptomes of RASFs isolated from multiple patient samples at progressing stages of pathogenesis.

## Conclusion

In summary, our first complete transcriptome analysis of synovial fibroblast RNA from patients with rheumatoid arthritis using RNA-seq has provided important insights into the transcriptional regulation of gene expression in RASFs. Further in-depth, follow-up analyses using large patient populations will be necessary to validate the alterations in transcriptional regulation reported in this study and to provide the resources necessary to elucidate the molecular mechanisms underlying the role of SFs in the pathogenesis of RA.

## Methods

### RNA sequencing

Human SF RNA from 2 healthy female donors and 2 adult female RA patients (Additional file 1) was purchased from Cell Applications, Inc. (San Diego, CA). SFs were first isolated from synovial tissue either from healthy control donors or from patients with RA and cultured for two passages prior to RNA isolation. Paired-end cDNA libraries were prepared for each sample and sequenced using the Illumina TruSeq RNA Sample Preparation Kit, as described previously [11,12]. Briefly, the cDNA libraries were quantified using a Biotek EPOCH spectrophotometer and checked for quality and size using a Bio-Rad Experion DNA 1K chip. The four cDNA libraries were each diluted to 6 pM and spiked with 1% phiX control to improve base calling while sequencing. A 6 pM dilution of phiX control sample was also prepared for analysis. Following the Illumina cBot and HiSeq protocols, the four libraries and the phiX control underwent cluster generation on a HiSeq PE flow cell v3 and were then sequenced using a HiScanSQ (Illumina). A paired-end (2×101) run was performed using the SBS Kit (Illumina). Real-time analysis and base calling were performed using the HiSeq Control Software Version 1.4.5 (Illumina). The resulting basecalling (.bcl) files were converted to. FASTQ files using Illumina’s CASAVA 1.8 software. The number of reads for each sample type was analyzed using the Student’s t-test in SigmaPlot version 11.0 (Systat Software Inc., San Jose, CA). A p-value of below 0.05 was considered significant. The sequence data have been submitted to the NCBI Short Read Archive with accession number SRA048057.1.

### Mapping of RNA-seq reads and transcript assembly and abundance estimation using Tuxedo Suite

Paired-end fastq sequence reads for each sample were aligned to the UCSC *Homo sapiens* reference genome hg19 using TopHat v1.3.0 [54,55] integrated with Bowtie v0.12.7 [56], as described previously [11,12]. The resulting aligned reads were analyzed further by Cufflinks v1.0.3 [55,57]. The aligned reads were assembled into transcripts, either with or without a reference genome, and the expression of those transcripts were reported in *Fragments Per Kilobase of exon per Million fragments mapped* (FPKM). Cuffdiff analysis was performed, with use of the reference genome, to determine differential expression of known isoforms between pooled RA patient samples and pooled control samples. To detect novel isoforms, Cufflinks was run without a reference genome. The RA and control transcript files were compared to the reference genome using Cuffcompare to filter out previously discovered transcripts. To test the differential expression of these novel isoforms, Cuffdiff analyses were performed using the combined transcript files as the reference genome. Cuffdiff analyses were performed two ways: comparing the RA patient transcripts to the control transcripts, using the RA patient transcripts as the reference genome; and comparing the RA patient transcripts to the control transcripts, using the control transcripts as the reference genome.

### Visualization of mapped reads

Aligned reads were visualized using a local copy of the Integrative Genomics Viewer (http://www.broadinstitute.org/igv/). The output files generated from TopHat were converted into files viewable in IGV by BEDTools [58] and then processed further by the “count” function in igvtools (included with the IGV software) to create an average alignment track viewable as a bar chart. The log_2_ of the frequency of the reads was plotted to better visualize the extensive range of the read coverage. Individual gene views were created by first merging the TopHat output files from the RA and control samples into two files using SAMTools [59]. These merged files were processed in the same way as above with the “count” function in igvtools. The raw frequency of the reads was visualized in this case.

### Automated literature search

Multiplex literature mining analysis was conducted with PubMatrix, [22] as described previously [60]. We restricted our search to human symbols approved by HUGO Gene Nomenclature Committee (HGNC) for the top 10 genes and isoforms for each category. Terms “rheumatoid arthritis”, “osteoarthritis”, “arthritis” and “disease” were used for cross-referencing candidate genes.

### Functional analysis of differentially expressed gene lists using ingenuity pathway analysis

The differentially expressed gene lists were submitted to Ingenuity Pathway Analysis (IPA) v9.0-3211 (Ingenuity Systems, Inc., Redwood City, CA). Genes with a two-fold or greater change in expression between the RA group and the control group were used. The settings for the core analysis were as follows: Ingenuity Knowledge Base; Endogenous Chemicals not included; Direct and Indirect relationships; molecules per pathway: 70; and networks per analysis: 25.

## Competing interests

The authors declare that they have no competing interests.

## Authors’ contribution

DPH and MG carried out the experimental studies and data analysis. DPH, DNG and LQZ participated in the design of the study and performed the statistical analysis. DPH drafted the manuscript. SQY conceived of the study, and participated in its design and coordination and helped to draft the manuscript. All authors read and approved the final manuscript.

## Supplementary Material

Additional file 1 RNA samples from synovial fibroblasts for RNA-seq analysis. Click here for file

Additional file 2 Top fifty up- and down- regulated genes expressed only in normal synovial RNA or only in rheumatoid arthritis synovial RNA. Click here for file

Additional file 3 PubMatrix Literature Search. Click here for file

Additional file 4 Top fifty up- and down- regulated genes expressed in rheumatoid arthritis synovial RNA. Click here for file

Additional file 5 Top fifty up- and down- regulated isoforms expressed only in normal synovial RNA or only in rheumatoid arthritis synovial RNA. Click here for file

Additional file 6 Top fifty up- and down- regulated known isoforms expressed in rheumatoid arthritis synovial RNA. Click here for file

Additional file 7 Top fifty up- and down- regulated novel isoforms expressed only in normal synovial RNA or rheumatoid arthritis synovial RNA. Click here for file

Additional file 8 Top fifty up- and down- regulated novel isoforms expressed in rheumatoid arthritis synovial RNA. Click here for file
