# Supplementary material for: RNA-seq analysis of synovial fibroblasts brings new insights into rheumatoid arthritis
Source: Cell Biosci. 2012 Dec 21;2:43. doi: 10.1186/2045-3701-2-43 (PMC3560277; doi:10.1186/2045-3701-2-43)
Supplement: Additional file 1 — RNA samples from synovial fibroblasts for RNA-seq analysis. [file 2045-3701-2-43-S1.docx]

Additional File 1: RNA Samples from Synovial Fibroblasts for RNA-Seq Analysis

Phenotype Age Race Sex Vendor Catalog Number Lot Number

WT 46 W F Cell Applications 408-R10a 2645

WT 73 W F Cell Applications 408-R10a 2571

RA 55 W F Cell Applications 408-RA-R10a 1990

RA 73 W F Cell Applications 408-RA-R10a 2708

WT = Wild Type: RA = Rheumatoid Arthritis; W = White, F = Female
